# Current Status and Future Trends for Modification Technology of Flame Retardant Nylon 66

**DOI:** 10.3390/polym17081074

**Published:** 2025-04-16

**Authors:** Bingtao Feng, Senlong Yu, Hengxue Xiang, Lili Li, Meifang Zhu

**Affiliations:** State Key Laboratory of Advanced Fiber Materials, College of Materials Science and Engineering, Donghua University, Shanghai 201620, China; 15832987969@163.com (B.F.); hengxuexiang@dhu.edu.cn (H.X.); lll@dhu.edu.cn (L.L.); zmf@dhu.edu.cn (M.Z.)

**Keywords:** nylon 66, flame retardant, blending, copolymerization, post finishing

## Abstract

Nylon 66 (PA66) has been widely used in automotive, electronics, textiles and other fields due to its excellent mechanical properties, chemical corrosion resistance and thermal stability. However, the fire hazard caused by its flammability severely limits its further application in high–end and high–risk fields. Therefore, improving the flame retardancy of PA66 to enhance its safety has become the focus of current research. This review aims to better understand the research status and development trends of flame retardant PA66. Firstly, the combustion process and flame retardant mechanism of PA66 were described. Secondly, the latest research progress of flame retardant PA66 was comprehensively reviewed, including blending, copolymerization and post–finishing flame retardant modification methods. Meanwhile, the research status of blending flame retardant PA66 was emphatically introduced, and the advantages and disadvantages of different additive flame retardants were analyzed. Finally, the future development direction of flame retardant PA66 is proposed, which provides an important reference for its follow-up study.

## 1. Introduction

With the rapid development of modern technology and industry, polymer materials have become an indispensable foundation for human society. Notably, engineering plastics have garnered significant attention due to their excellent properties and wide application prospects. Among various engineering plastics, nylon 66 (PA66), as an important polymer material, has been extensively utilized in diverse fields due to its excellent mechanical properties, thermal stability and chemical corrosion resistance [1,2,3,4]. In recent years, with the advancement of science and technology, the popularity of petrol–electric vehicles, smart homes and electronic products has led to a dramatic increase in the demand for PA66. At the same time, potential safety hazards also follow. The devastating impact of fires led to a substantial loss of life and property, which has driven increased societal focus on the flame retardancy of materials. However, the flame retardancy of PA66 is poor. For example, the limiting oxygen index (LOI) is between 21% and 24%, and the UL94 flame retardant grade is V–2, which is a combustible material [5,6,7]. In addition, PA66 will generate a significant amount of combustible droplets during combustion, which will further expand the fire hazard [8,9]. Therefore, to enhance the safety performance of PA66 and expand its application field, the research and development of flame retardant PA66 is particularly important.

In the research of flame retardant PA66, researchers have explored a variety of flame retardant technologies and modification methods. From traditional additive flame retardants to reactive flame retardants [10,11,12,13,14,15,16] and nanocomposite flame retardant systems [17,18], each technological advancement has brought new breakthroughs in material properties. These studies not only enrich the theoretical system of flame retardant materials but also expand their practical application possibilities. However, the research of flame retardant PA66 still faces many challenges [19]. A critical issue that researchers must solve is how to achieve effective flame retardancy while maintaining the original excellent properties of PA66 [20]. At the same time, with increasing environmental awareness, the development of environmentally friendly, non–toxic and efficient flame retardants has also become a hot and difficult research issue.

With the increasing attention to safety performance around the world, countries worldwide have introduced stricter flame retardant standards and regulations, imposing higher requirements for advanced research and development in flame retardant PA66. Therefore, a comprehensive understanding of the research status, development trends and challenges of flame retardant PA66 is essential for promoting its wider use in practical applications. In industrial production, flame retardant PA66 is predominantly prepared through the blending method [4]. Therefore, this article focuses on the research progress of flame retardant PA66 prepared by the blending method and discusses the latest achievements of the flame retardant mechanism, performance characterization, and other aspects. Through comparing and analyzing the advantages and limitations of different flame retardant systems, this article provides a valuable reference for the future research and development of flame retardant PA66.

## 2. Flame Retardant Mechanism of Flame Retardants

### 2.1. The Combustion Mechanism of PA66

**Figure 1:**
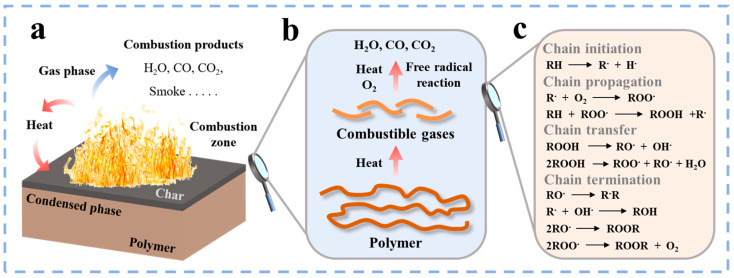
(**a**,**b**) The combustion process of the polymer; (**c**) the combustion mechanism of PA66 [24].

Early studies indicated a significant correlation between the thermal decomposition process of polymers and their fire hazards. Purser [21] systematically evaluated the toxicity of combustion products and elucidated the critical role of polymer thermal decomposition products in fire development, thereby establishing an important theoretical foundation for subsequent research on polymer combustion mechanisms. The combustion behavior of PA66 resembles that of most polymers, involving three essential components: oxygen, ignition source and combustible material. Under the action of air or oxygen, the ignition source provides heat, increasing the temperature of the polymer and initiating its degradation, leading to the breakdown of chemical bonds. When numerous chemical bonds are broken, degradation products are produced, some of which become combustible gases. These combustible gases accelerate the combustion of the polymer in the presence of oxygen, thereby releasing more heat. When the heat is sufficient, the polymer will undergo thermal decomposition in the condensed phase. This process leads to further accumulation of combustible materials on the polymer surface, whose subsequent combustion enhances the heat transfer to the interior of the polymer, thus causing a larger flame [22,23,24]. The combustion cycle mechanism of the polymer is shown in Figure 1a,b.

The thermal oxidative degradation of PA66 is a complex physical and chemical process involving many reaction mechanisms and conditions. The process typically occurs in the presence of oxygen and at high temperatures, comprising four key steps: chain initiation, chain growth, chain transfer and chain termination. The combustion mechanism is shown in Figure 1c. Under the combined influence of high temperature and oxygen presence, the C–H bond and C–C bond in the PA66 molecular chain are first broken, and the alkyl radical (R·) is formed. These R· subsequently react with oxygen, resulting in the formation of peroxide radicals (ROO·). The ROO· further reacts with the PA66 molecule to form hydroperoxide (ROOH). ROOH is unstable at high temperatures and will further decompose to produce more free radicals. Finally, these free radicals terminate the chain reaction through coupling or disproportionation reactions to form a stable product. This series of reactions not only revealed the complex chemical changes during PA66 combustion but also provided a theoretical foundation for the flame retardant modification of PA66 [24,25].

### 2.2. The Flame Retardant Mechanism of PA66

The combustion of polymers requires heat, combustibles and oxygen [26], and the flame retardant can be achieved by slowing or preventing one or more of them. At present, the flame retardancy of PA66 is primarily achieved through isolating the exchange of heat and oxygen, interrupting the combustion chain reaction, etc. It mainly includes forming a protective layer on the burning surface to insulate oxygen and heat, absorbing the heat from the combustion zone, producing noncombustible gases to reduce flammable gases and oxygen concentrations, and capturing the free radicals involved in the combustion process. There are four main flame retardant mechanisms:

#### 2.2.1. Gas Phase Flame Retardant Mechanism

Gas phase flame retardancy is achieved by interrupting combustion or terminating the chain reaction in the gas phase. For example, when the polymer is burned, some flame retardants will decompose and release significant amounts of inert gases. These gases not only dilute the concentration of combustible gases and oxygen but also absorb heat from the burning surface during their release, thereby achieving the purpose of flame retardant [12]. Some flame retardants will produce high–density water vapor when heated, which will cover the combustion surface of PA66 and prevent the contact between decomposed combustibles and oxygen, thus promoting the self–extinguishing of the combustion surface. Additionally, some flame retardants will release reactive free radicals, which will capture the free radicals generated during PA66 combustion, thereby interrupting the combustion reaction [27].

#### 2.2.2. Condensed Phase Flame Retardant Mechanism

Condensed phase flame retardancy works through heating the flame retardant to form a dense protective char layer on the PA66 surface, thereby achieving the purpose of flame retardant. The protective char layer acts as a barrier, blocking the heat transport and combustibles exchange between PA66 and the outside world. This mechanism retards the combustion reaction, reduces smoke and exhaust gases in the rising stage of fire, and gives full play to the flame retardant effect of flame retardants in the condensed phase [28]. Moreover, some flame retardants can also inhibit the production of high–energy free radicals and limit the escape of combustible gases through the reaction, further enhancing the flame retardancy.

#### 2.2.3. Synergistic Flame Retardant Mechanism

Synergistic flame retardancy refers to two or more flame retardants working together for PA66, which makes PA66 have the characteristics of condensed and gas phase flame retardants at the same time [29]. The synergistic flame retardant system demonstrates superior performance compared to a single flame retardant because different flame retardants can synergistically inhibit the combustion of PA66. At present, the synergistic flame retardant systems applied to PA66 mainly include phosphorus–nitrogen flame retardant and organic–inorganic flame retardant, etc. [30,31].

#### 2.2.4. Interrupting Heat Exchange Flame Retardant Mechanism

Interrupted heat exchange flame retardancy operates by transferring the heat from the combustion zone of PA66; this approach makes the temperature of PA66 lower than its pyrolysis temperature, thereby achieving the flame retardant effect. For example, a synergistic flame retardant system consisting of Sb_2_O_3_ and melamine cyanurate (MCA) was utilized to enhance the flame retardancy of PA66. When burned by heat, the synergistic system can take away significant amounts of heat by melting and dripping, reducing the heat transferred to PA66, thereby effectively inhibiting the combustion [32].

## 3. The Development of Flame Retardant PA66

**Figure 2:**
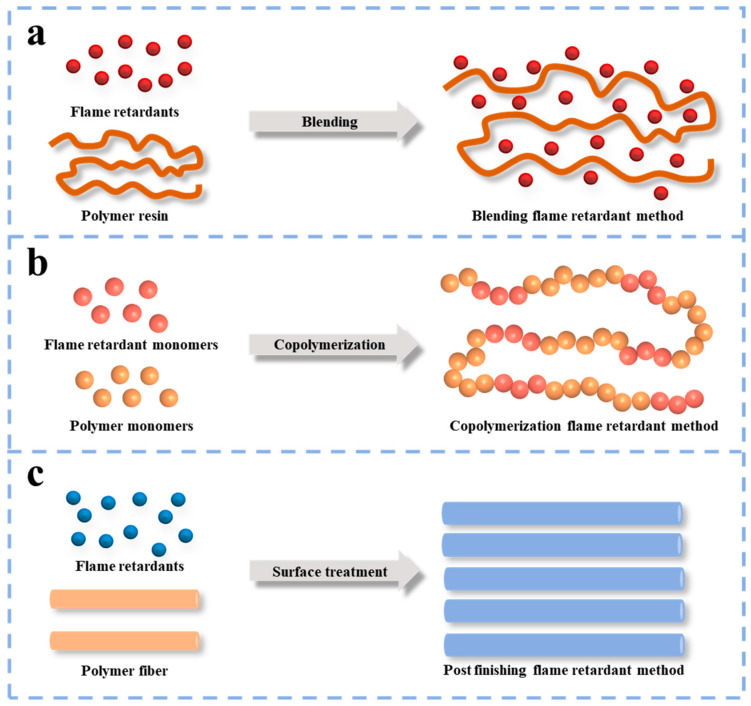
The flame retardant methods of PA66 [24]: (**a**) blending flame retardant modification, (**b**) copolymerization flame retardant modification; (**c**) post–finishing flame retardant modification.

PA66 is a combustible material, which makes it unable to meet the requirements of flame retardant in certain specialized fields. Therefore, how to prepare flame retardant PA66 efficiently and simply has become a hot spot of current research. At present, the flame retardant methods for PA66 mainly include blending flame retardant modification (Figure 2a), copolymerization flame retardant modification (Figure 2b) and post–finishing flame retardant modification (Figure 2c) [24]. These methods have their characteristics, aiming to improve the flame retardancy of PA66 during application, ensuring the material meets safety requirements in different fields.

### 3.1. Blending Flame Retardant Modification

Blending flame retardant modification is achieved by mechanically blending flame retardants with PA66. This method is simple to operate and widely applicable, and it is the most common method used in industry [33]. In the blending flame retardant modification, the earliest flame retardants are predominantly halogen flame retardants, which have excellent flame retardancy. However, these halogen flame retardants will release toxic and harmful hydrogen halide gases during application, which not only damage the ecological environment but also harm human health; this contradicts the principles of green and sustainable development. The application of halogen flame retardants in daily production and life has been greatly limited [34,35]. As a result, the development of halogen–free flame retardants has gradually become the main research direction of flame retardant.

#### 3.1.1. Phosphorus Flame Retardants

**Figure 3:**
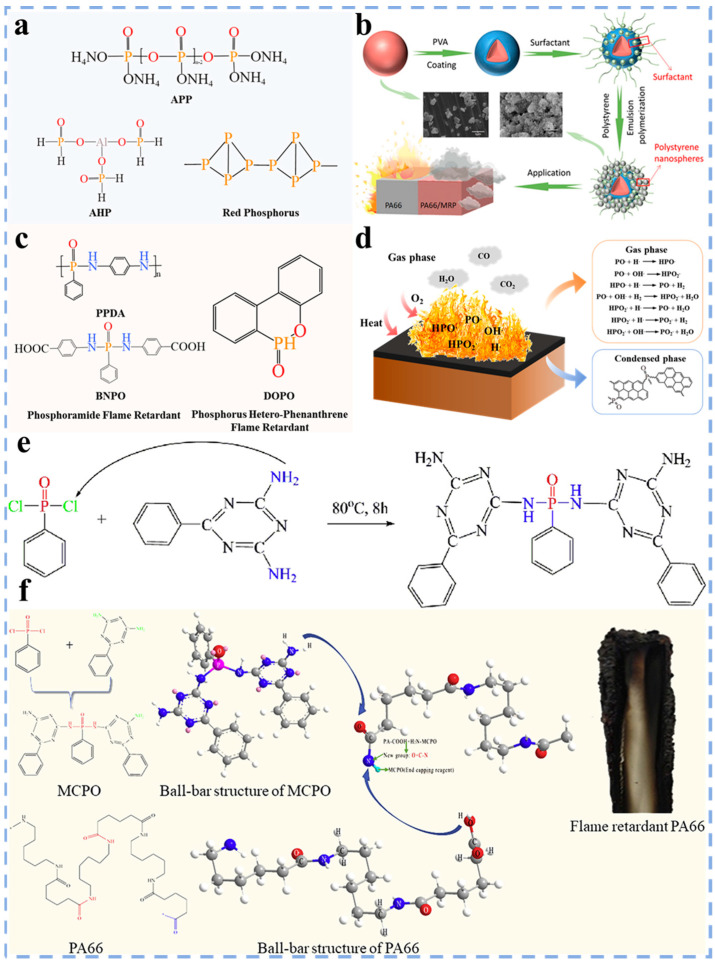
(**a**) Inorganic phosphorus flame retardants; (**b**) synthetic route of flame retardant PA66 [38]; (**c**) organic phosphorus flame retardants; (**d**) the flame retardant mechanism of phosphorus flame retardants [43]; (**e**) the synthetic routes of MCPO and (**f**) the end capping route of flame retardant PA66 [35].

Phosphorus flame retardants have become an ideal alternative to halogen flame retardants owing to their unique flame retardant mechanism and low toxicity. Depending on the different chemical structures and compositions, phosphorus flame retardants can be classified into two main categories: inorganic and organic phosphorus flame retardants. The commonly used inorganic phosphorus flame retardants include ammonium polyphosphate (APP), aluminum hypophosphite (AHP) and red phosphorus (Figure 3a). As a classic representative of inorganic phosphorus flame retardant, red phosphorus will produce P_2_O_5_ in the combustion process. The P_2_O_5_ will quickly transform into phosphoric anhydride and water–soluble inorganic acid by absorbing water. These substances can effectively reduce flame height and heat release rate. Moreover, under high temperatures, red phosphorus can catalyze the formation of a refractory coke layer, which can form a thermal barrier on the combustion surface and prevent further transfer of oxygen and heat [36,37].

However, inorganic phosphorus flame retardants face several limitations, including high moisture absorption, poor compatibility with polymers and easy thermal decomposition, which makes them difficult to be directly used for flame retardant modification of polymers. Therefore, it is essential to pretreat inorganic phosphorus flame retardants before use. Chen et al. [38] coated red phosphorus with an inner polyvinyl alcohol (PVA) layer and an outer polystyrene nanosphere (PSNS) layer (Figure 3b), significantly improving the properties of red phosphorus. The study found that the ignition point of red phosphorus is 260 °C, indicating its poor thermal stability. After modification with PSNS, the ignition point of red phosphorus increased by nearly 200 °C, and the thermal stability was significantly enhanced. Simultaneously, the moisture absorption rate of the modified red phosphorus decreased by 40 times. When 5 wt% of PSNS@RP was added to PA66, the LOI of PA66 increased from 24.8% to 34.2%, reaching the UL94 V–0 flame retardant standard, showing excellent flame retardancy.

Organic phosphorus flame retardants are different from inorganic phosphorus flame retardants. It is a kind of phosphorus compound containing organic groups, which has the advantages of good dispersibility and low required dosage. According to the differences in phosphorus structure, organic phosphorus flame retardants can be classified into phosphoramide flame retardants, phosphorus hetero–phenanthrene flame retardants, etc. (Figure 3c) [33,39,40]. In the field of flame retardant mechanism research, Camino et al. [41] conducted a study in 1984 on the thermal degradation behavior of the polyphosphate-pentaerythritol mixtures, laying an important foundation for revealing the mechanism of organic phosphorus flame retardants. Current research indicates that organic phosphorus flame retardants primarily achieve flame retardancy through gas phase and condensed phase flame retardant mechanisms. The specific mechanisms include: (1) In the combustion process, organic phosphorus flame retardants produce free radicals such as HPO· and PO·, which can trap the H· and OH· groups, thus inhibiting the combustion reaction; (2) Under the condition of high temperature, organic phosphorus flame retardants can catalyze the dehydration of C, H and O elements to form a dense char layer. Concurrently, it will produce phosphate on the surface of PA66, isolating the contact between PA66 and air, thereby achieving the flame retardant PA66 (Figure 3d) [42,43]. Lyu et al. [35] prepared bis–*N*–benzoguanamine–phenylphos–phamide (MCPO) (Figure 3e) and melt–blended PA66 and MCPO using a twin–screw extruder. Research has found that the –NH_2_ of MCPO can undergo dehydration reaction with –COOH at the end of PA66 main chains, thereby grafting flame retardant elements such as benzene rings, P, and triazine rings onto the tail of PA66 main chains (Figure 3f). When the MCPO content was 8 wt%, the flame retardant grade of PA66 reached UL94 V–0, indicating that this modification method can significantly enhance the flame retardancy of PA66. Meanwhile, 9,10–dihydro–9–oxa–10-phosphaphenan–threne–10–oxide (DOPO) and its derivatives have also gradually become the research hotspot of phosphorous flame retardants. Xie et al. [33] synthesized a star–shaped DOPO derivative (GL–3DOPO) using glycerol, acryloyl chloride and DOPO. The study found that the thermal decomposition temperature of DOPO before modification was 194 °C (under a N_2_ atmosphere). The thermal decomposition temperature of modified GL–3DOPO increased to 360 °C (under a N_2_ atmosphere). When the addition amount of GL–3DOPO was 25 wt%, the flame retardant grade of PA66 reached UL94 V–0, and the LOI was significantly improved. Although organic phosphorus flame retardants are widely used, they also face challenges, such as poor polymer compatibility and reduced mechanical properties of polymers. To overcome these limitations, researchers have increasingly focused on exploring alternative flame retardants. Among these, nitrogen flame retardants have attracted much attention owing to their unique flame retardant mechanism and environmental characteristics.

#### 3.1.2. Nitrogen Flame Retardants

Nitrogen flame retardants are mainly used to achieve the purpose of flame retardant through the gas phase mechanism. When heated, they will decompose to produce N_2_, NO_2_, NH_3_ and other noncombustible gases. These gases will not only take away the heat from the surface of PA66 but also dilute the concentration of oxygen and flammable gases so that the combustion reaction is inhibited.

**Figure 4:**
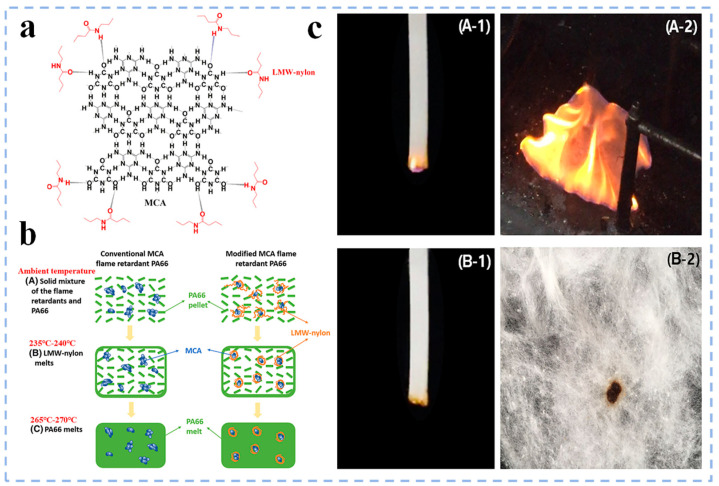
(**a**) Intermolecular hydrogen bonding between modified resin and MCA; (**b**) flow and dispersion processes of MCA before and after modification; (**c**) UL 94 testing photos of MCA/PA66 before modification (**A–1** and **A–2**) after modification (**B–1** and **B–2**) [44].

Currently, commonly used nitrogen flame retardants mainly include melamine (MC), MCA, etc. Among them, researchers have conducted many studies on MCA due to its good flame retardancy. Luo et al. [44] wrapped MCA with low molecular weight nylon (Figure 4a), which significantly improved the flowability and dispersion of MCA in PA66 (Figure 4b). It was found that when 10 wt% modified MCA was added, the flame retardancy of PA66 was significantly improved compared with the conventional MCA/PA66 system, reaching UL94 V–0 level (Figure 4c). In terms of mechanical properties, compared with pure PA66, the tensile strength and Izod notched impact strength of conventional MCA/PA66 decreased by 8.6% and 38.4%, respectively, while the modified MCA/PA66 only decreased by 0.66% and 14.4%. The results show that this modified method can effectively maintain the mechanical properties of PA66 while improving the flame retardancy. However, nitrogen flame retardants exhibit low flame retardant efficiency, requiring high quantities to achieve a good flame retardant effect when used alone. To overcome this limitation, nitrogen flame retardants are often combined with organic phosphorus compounds, forming synergistic phosphorus–nitrogen systems.

#### 3.1.3. Phosphorus–Nitrogen Flame Retardants

**Figure 5:**
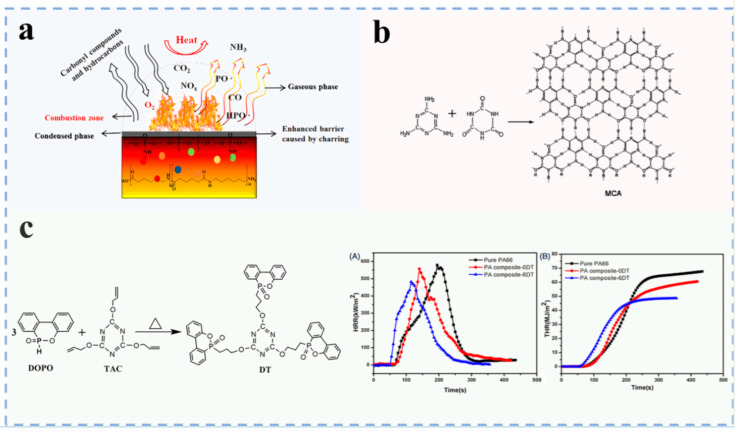
(**a**) The flame retardant mechanism of PA composites [45]; (**b**) self–assembly of the MCA [5]; (**c**) preparation route of DT. Different heat release curves of PA66 and its composites during combustion: (A) heat release rate, (B) total heat release [47].

Phosphorus–nitrogen flame retardants mainly include phosphorus–nitrogen synergistic flame retardants, phosphorus–nitrogen intumescent flame retardants, etc. They primarily consist of three components: an acid source (phosphorus–containing compounds), a carbon source (high–carbon polyhydroxy compounds) and a gas source (nitrogen–containing compounds), corresponding to the three flame retardant elements of P, N and C. When phosphorus–nitrogen flame retardants are heated and burned in PA66, the carbon source and PA66 will dehydrate under the action of acid sources (phosphoric acid, polyphosphoric acid, polymetaphosphoric acid, etc.), resulting in the formation of a dense and noncombustible char layer. This can isolate oxygen and heat exchange between the interior and exterior of the char layer. In addition, the decomposition of the gas source releases nonflammable gases (e.g., NH_3_ and N_2_), which reduce the concentration of both flammable gases and oxygen on the PA66 surface. When the interior of the char layer formed on the combustion surface of PA66 does not receive sufficient heat and oxygen, combustion will be interrupted, thereby enhancing the flame retardancy of PA66 [45]. The flame retardant mechanism of phosphorus–nitrogen flame retardant is shown in Figure 5a.

At present, phosphorus–nitrogen flame retardants are extensively used in polymers to enhance their flame retardancy, and researchers have conducted much research on phosphorus–nitrogen flame retardants. Liu et al. [5] prepared melamine cyanurate–microencapsulated red phosphorus flame retardant by the molecular self–assembly method. They found that PA66 containing flame retardant has excellent flame retardancy and mechanical properties (Figure 5b). Chen et al. [46] employed a combination of aluminum phosphinate (OP) and melamine polyphosphate (MPP) to achieve synergistic enhancement of flame retardancy in PA66 composites. The study revealed that the addition of OP promoted the uniform dispersion of flame retardant particles in the composites. OP and MPP exhibited significant synergistic effects. When the dosage of flame retardant was 16 wt%, the composite achieved a UL94 V–0 flame retardant grade with an LOI of 33.5%. Guo et al. [47] synthesized a phosphorus–nitrogen flame retardant (DT) through a DOPO and 2,4,6–triellyloxy–1,3,5–triazine (TAC) addition reaction, and PA composites were prepared by mixing PA66, DT and polyphenylene ether (PPO). PA composites have excellent flame retardancy, achieving an LOI of 29.0%. The total heat released and the effective combustion heat are reduced by 27% and 21%, respectively, compared to pure PA66 (Figure 5c).

Although phosphorus–nitrogen flame retardants can significantly improve the flame retardancy of PA66, they also have certain limitations. For instance, some phosphorus–nitrogen flame retardants readily decompose at high temperatures, leading to reduced flame retardant efficiency. Additionally, some phosphorus–nitrogen flame retardants may impair the physical properties (strength and toughness) of PA66. Based on the disadvantages of phosphorus–nitrogen flame retardants, the development and application of organic–inorganic flame retardants have become an effective solution.

#### 3.1.4. Organic–Inorganic Flame Retardants

Organic–inorganic flame retardants are synthesized by compounding inorganic flame retardant components with organic flame retardant components. This type of flame retardant combines the advantages of both organic and inorganic flame retardants. In recent studies on flame retardant modification of PA66, the application of organic–inorganic flame retardants has gradually attracted attention. The commonly used organic–inorganic flame retardants primarily include organic nitrogen–based inorganic flame retardants and organic phosphorus–based inorganic flame retardants. In organic–inorganic flame retardants, inorganic components such as clay, montmorillonite, sepiolite, nano–silica and magnesium oxide are widely used. Sheng et al. [48] synthesized an imidazolium organoclay (I–Clay) to improve the flame retardancy of PA66. The research results showed that the heat release rate of PA66 with 2 wt% I–Clay was reduced by 36% compared to pure PA66, indicating that I–Clay could improve the flame retardancy of PA66. Qin et al. [49] prepared a nanocomposite of PA66 and montmorillonite. The study found that when the amount of alkylammonium–modified montmorillonite was 5 wt%, the heat release rate of the composite material was reduced by 59% compared to pure PA66, indicating that alkylammonium–modified montmorillonite can significantly enhance the flame retardancy of PA66.

For the application of organic nitrogen–based inorganic flame retardants in PA66, Liu et al. [50] used MCA for the surface modification of magnesium hydroxide (MH), and the study showed that MCA–modified MH significantly enhanced the mechanical properties of PA66 compared to the MH modified by a silane coupling agent. Further study found that when the ratio of MCA/MH is 35/65, the LOI of PA66 reached its highest value of 46%.

**Figure 6:**
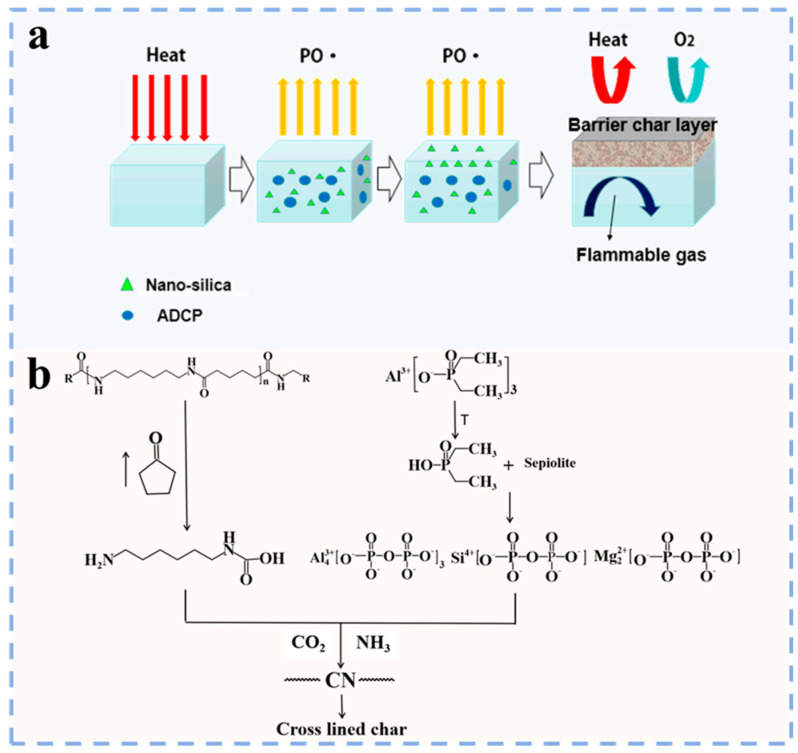
(**a**) Analysis of flame retardant mechanism [distributed under the terms and conditions of the Creative Commons Attribution (CC BY) license http://creativecommons.org/licenses/by/4.0/ (accessed on 7 March 2025), from [51]; (**b**) thermal degradation mechanism of PA66/AlPi/sepiolite [31].

On the other hand, the application of organic phosphorus–based inorganic flame retardants in PA66 has garnered significant attention. Zhang et al. [51] co–modified PA66 by using dicyclohexenyl aluminum hypophosphite (ADCP) and nano–silica. They observed that ADCP facilitated the development of a porous carbon layer on the PA66 combustion surface, while nano–silica exhibited surface migration tendencies, improving the oxidation resistance and structural density of the carbon layer. This enhanced carbon layer structure effectively inhibits the transfer of heat, oxygen and flammable gases, thus significantly improving the flame retardancy of PA66 (Figure 6a).

Similarly, Zhan et al. [31] also studied the application of organic phosphorus–based inorganic flame retardants for enhancing the flame retardancy of PA66. They first used sepiolite and aluminum diethylphosphinate (AlPi) for synergistic flame retardant modification of PA66 and found that when the addition of AlPi/sepiolite was only 10 wt%, PA66 showed good flame retardancy. The addition of sepiolite catalyzes the degradation of AlPi, altering the degradation pathway of the PA66/AlPi system. This process generates diethylphosphinic acid, magnesium phosphate and silicon phosphate (Figure 6b), thereby enhancing the char yield and thermal stability of PA66 at high temperature. Then, Zhan et al. [52] further studied the synergistic flame retardant effect of AlPi and nano–silica. The study indicated that higher nano–silica loading led to a continuous enhancement in the flame retardancy of the PA66 composite. When the dosage of nano–silica reaches 5 wt%, the PA66 demonstrates an LOI of 37.3% along with a UL94 V–0 flame retardant grade. This effect is primarily attributed to the formation of a thicker and more solid char layer on the PA66 surface by nano–silica, which significantly inhibits heat transfer and volatile release, thus enhancing the flame retardancy of PA66. In addition, Zhan et al. [53] also studied the synergistic flame retardant effect of AlPi and micron–sized magnesium oxide (MgO) in PA66. It was found that a total loading of 10 wt% AlPi and MgO significantly enhanced the flame retardancy of PA66. Specifically, MgO promotes the degradation of AlPi, resulting in the generation of magnesium phosphate that effectively traps flame retardant elements in the condensed phase. Additionally, the catalyzed charring of magnesium phosphate enhances the char layer formation on the PA66 surface, effectively protecting PA66 from the influence of oxygen and heat.

At present, the method of blending PA66 with flame retardant by twin–screw extruder has been widely used in the flame retardant modification of PA66. Although this method can simply and effectively enhance the flame retardancy of PA66, it will also affect other properties of PA66. For example, when preparing flame retardant PA66 through blending, a substantial amount of flame retardants is generally required to ensure effective flame retardancy. However, due to the problem of compatibility, the large additional amount will cause the flame retardant to migrate and precipitate during both the processing and application of PA66. Consequently, the mechanical properties of PA66 are adversely affected [54]. For this reason, the researchers proposed a novel concept of copolymerized flame retardant, which made up for the defects of the blending method and solved the problem of reduced mechanical properties.

### 3.2. Copolymerization Flame Retardant Modification

Copolymerization flame retardant modification is achieved by grafting flame retardants to the macromolecular chains of PA66. This not only avoids compatibility issues between flame retardants and PA66, ensuring long–lasting flame retardancy, but also maintains the thermal properties and mechanical and processing performance of PA66, which can better meet the performance requirements of PA66 in practical applications [55]. At present, the reactive flame retardants used in PA66 mainly include phosphorous flame retardants, nitrogen flame retardants and phosphorous–nitrogen flame retardants.

**Figure 7:**
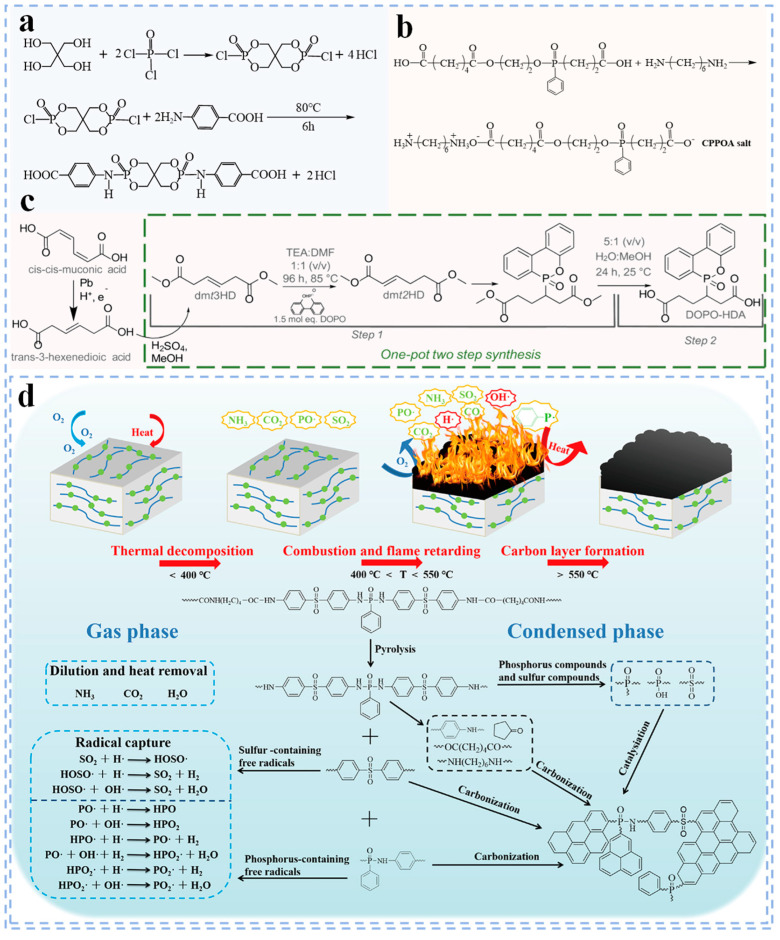
(**a**) The synthetic route of TRFR [12]; (**b**) route for synthesis of CPPOA salt [15]; (**c**) synthesis route of DOPO–HDA [16]; (**d**) schematic diagram of flame retardant mechanism of PA66–PDDDS [14].

Researchers have conducted extensive studies on copolymerized flame retardant PA66 (Table 1). Fu et al. [12] developed a “trinity” reactive flame retardant (TRFR) by synthesizing phosphorus oxychloride (POC), pentaerythritol and *p*–aminobenzoic acid. Subsequently, they prepared flame retardant PA66 by polymerizing TRFR with PA66 salt (Figure 7a). It was observed that even a small quantity of TRFR salt can significantly improve the flame retardancy of PA66. This is because TRFR will decompose during the combustion of PA66, generating compounds containing C, P and N. These compounds can dehydrate the combustion surface to form char and produce noncombustible gases, thereby forming a dense, porous and noncombustible char layer. This char layer can serve to isolate heat and oxygen, thereby preventing the continuous combustion of PA66.

In order to make PA66 have excellent flame retardancy while maintaining good mechanical properties, Zhang et al. [15] first prepared CPPOA salt by the reaction of 4–(2-(((2–carboxyethyl)(phenyl)phosphoryl)oxy)ethoxy)–4–oxohexanoic acid (CPPOA) with 1,6–diaminohexane (Figure 7b). Subsequently, the CPPOA salt was copolymerized with PA66 salt to produce intrinsic flame retardant PA66. The research results show that when the CPPOA content is 6 wt%, PA66 exhibits both excellent flame retardancy and satisfactory mechanical performance. Carter et al. [16] synthesized DOPO–grafted diacid (DOPO–HDA) through one–pot isomerization of dimethyl–trans–3–hexenedioate (dmt3HD) and Michael–addition (MA) reaction. Then, they copolymerized DOPO–HDA with adipic acid and hexamethylenediamine to prepare flame retardant PA66 (Figure 7c). The study found that the introduction of DOPO–HDA enhances the flame retardancy of PA66 while significantly preserving its other essential properties. Wu et al. [14] innovatively designed and synthesized P–phenyl–*N*,*N*′–bis(p–sulfanilylphenyl) (PDDDS) containing sulfur and phosphorus flame retardant elements and introduced it into PA66 through polycondensation. Research has found that adding just 2% PDDDS to PA66 significantly improves its flame retardancy, mechanical properties and heat resistance. The excellent flame retardancy of PA66 is primarily attributed to the decomposition of PDDDS into phosphate and sulfur–containing compounds when heated, which promote the dehydration of PA66, leading to the formation of a dense and highly graphitized continuous char layer. Furthermore, the gas phase P–O and SO_2_ generated during the PDDDS decomposition process can effectively capture active free radicals (e.g., H· and OH·), thereby blocking the combustion reaction (Figure 7d).

Although copolymerized flame retardant PA66 exhibits excellent flame retardant effects and mechanical properties, this method still faces many challenges in practical applications. Due to the strict requirements for production equipment and complex synthesis steps, many technical obstacles need to be overcome to achieve large–scale industrial production. Therefore, although this method has shown great potential in laboratory research, there is still a difficult exploration path before it can be applied in practical industrialization.

### 3.3. Post–Finishing Flame Retardant Modification

The blending and copolymerization methods are mainly used for flame retardant modification of polymer resins, while for fibers and fabrics, post–finishing methods are often used for flame retardant modification. The post–finishing method adsorbs, precipitates, or chemically binds adhesives or flame retardants to the surface of fibers and fabrics by physical or chemical means [63]. This method is one of the earliest technologies applied to flame retardant modification and has become quite mature. The post–finishing method has few requirements for flame retardants, low production cost and a simple machining process. Thus, it remains one of the most prevalent flame retardant modification techniques utilized in industrial production.

**Figure 8:**
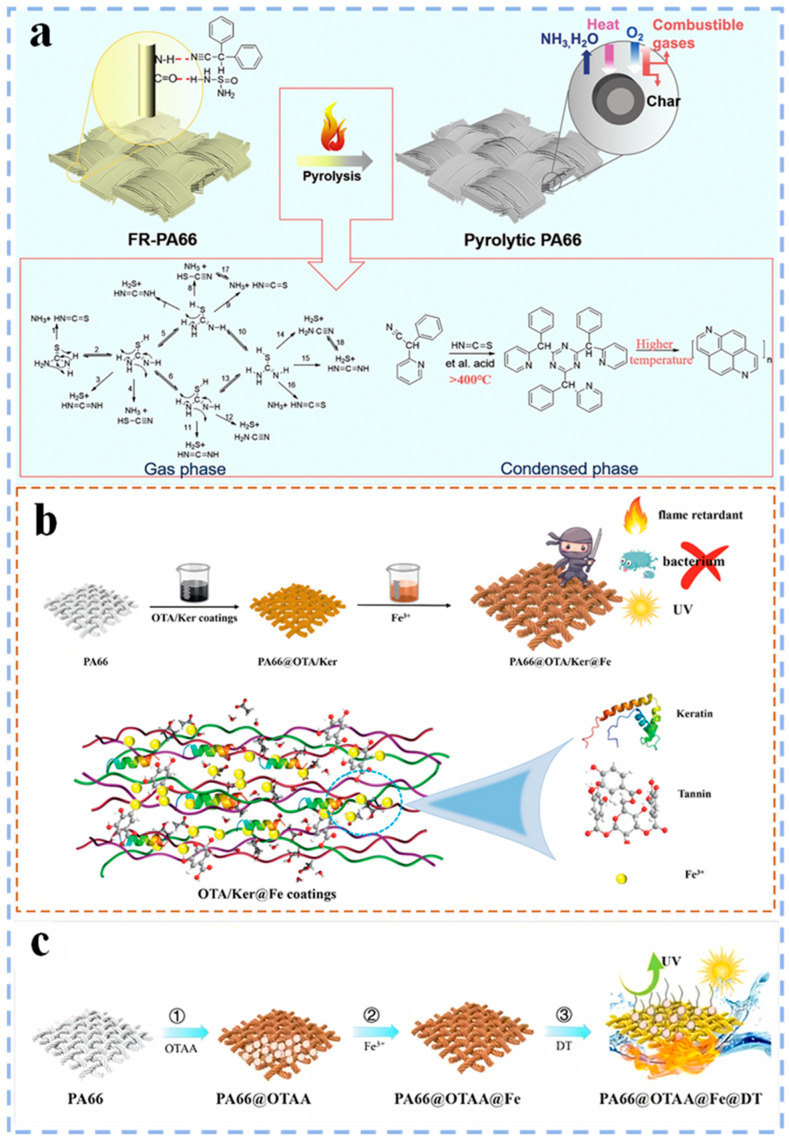
(**a**) Flame retardant mechanism of PA66–SPI–thiourea [64]; (**b**) process flow for preparing PA66@OTA/Ker@Fe [66]; (**c**) process flow for preparing PA66@OTAA@Fe@DT [67].

For the flame retardant modification of PA66 fiber and fabric, researchers have carried out a lot of studies by using the post–finishing method (Table 2). For example, Meng et al. [64] prepared flame retardant PA66 fabric by applying a coating of soybean protein isolation and thiourea onto the surface of PA66 fibers using a pad–dry method. The results showed that the presence of protein/thiourea not only eliminated the dripping phenomenon of PA66 fabric but also significantly improved its flame retardancy. This is due to the release of H_2_S, NH_3_ and H_2_O gases from the protein/thiourea composite coating during the combustion of PA66, which dilutes the concentration of flammable gases and oxygen. Additionally, the unsaturated double bond (such as –C=N) in the composite coating promotes the formation of a dense char layer, which effectively inhibits the combustion of PA66 (Figure 8a). In addition, Kundu et al. [65] utilized the sol–gel method to deposit a DOPO–alkoxysilane coating onto PA66 fabric, leading to a notable enhancement in its flame retardancy. Research shows that DOPO exhibits a quenching effect in the vapor phase, while Si compounds accelerate the limited charring of DOPO in the condensed phase and improve the thermal stability of char residues at high temperatures, thereby further enhancing the flame retardancy of PA66 fabric.

With the continuous pursuit of material safety and application diversification, the demand for PA66 performance is also increasing. The complex requirements of modern industrial production not only require PA66 to have excellent flame retardancy but also expect it to show outstanding versatility. To meet these needs, Jiang et al. [66] successfully prepared a multifunctional PA66 fabric by co–depositing keratin (Ker) and oxidized tannin (TA) on the surface of PA66 fabric and then chelating with Fe^3+^(PA66@OTA/Ker@Fe) (Figure 8b). The results show that PA66@OTA/Ker@Fe has good flame retardancy, achieving an LOI of 28.5% and meeting the UL94 V–0 standard. The fabric also shows excellent UV resistance and mechanical and antibacterial properties. In addition, Jiang et al. [67] also prepared another PA66 fabric with superior flame retardancy, super–hydrophobicity and UV resistance. This was achieved through coating PA66 fabric with tannins (TA) and L–Cysteine, then chelating it with Fe^3+^ and reacting with 1–dodecanethiol (DT) (PA66@OTAA@Fe@DT) (Figure 8c). This study provides an environmentally friendly and widely applicable strategy for the multifunctional modification of polyamide fabrics.

**Table 2:** Post–finishing flame retardant PA66.

Flame Retardants	Modification Method	References
Vinyltrimethoxysilane (VTMS)	Microwave grafting	[68]
Chitosan (CS), phytic acid (PA) and oxidized sodium alginate (OSA)	Deposit	[69]
Bio–based polyelectrolytes, CS, PA, (3–aminopropyl) triethoxysilane (APTES) and boron–doped APTES	Deposit and sol–gel treatment	[70]
Graphene oxide functionalized bio–macromolecule	One–pot deposition	[71]
CS and PA	Layer–by–layer assembly	[72]
CS, phosphorylated chitosan (PCS), and poly–acrylate sodium (PAS)	One–pot and layer–by–layer assembly	[73]
PA and Al^3+^	Layer–by–layer deposition technology	[74]
CS and phytic acid ammonia (PAA)	Pad–dry–cure technique	[75]

## 4. Conclusions and Prospect

With the rapid development of modern technology and industry, the demand for PA66 materials continues to increase. However, PA66 is a combustible material, and its widespread use raises safety hazards. The loss of life and property caused by fires has made society increasingly concerned about the flame retardancy of PA66. The combustion of PA66 requires elements such as heat, combustibles and oxygen; flame retardancy can be achieved by inhibiting one or more of these elements. At present, the flame retardancy of PA66 is mainly realized through forming a protective layer of thermal insulation and oxygen isolation on the combustion surface, absorbing heat from the combustion surface, producing noncombustible gases to dilute the concentration of oxygen and combustible gases, and interrupting combustion chain reactions.

The preparation methods of flame retardant PA66 mainly include the blending method, the copolymerization method and the post–finishing method. The production of flame retardant PA66 resin primarily employs blending and copolymerization methods, while the post–finishing method is mainly used for PA66 fibers and fabrics. At present, the blending method is the most widely used in industry. Commonly used flame retardants include phosphorus flame retardant, nitrogen flame retardant, phosphorus–nitrogen flame retardant and organic–inorganic flame retardant, etc. While these flame retardants are widely used, there are also some challenges, such as their poor compatibility with polymers, ease of decomposition at high temperatures, etc. These problems will have a certain impact on the performance of PA66. To address the limitations of current flame retardants and meet the high–performance requirements of PA66 in industrial applications, it is essential to develop new flame retardants. In response to the existing problems of flame retardants, the future development of flame retardant PA66 is expected to focus on the following key areas:

(1) High efficiency: With the continuous progress and enrichment of coating, microencapsulation, masterbatch and other technical means, the development of efficient flame retardants not only reduces the adverse impact on the original properties of PA66 but also significantly decreases the amount of flame retardants added, thereby leading to lower material costs. However, achieving high efficiency of flame retardants still requires continuous optimization and improvement of related technologies, such as further enhancing the performance and competitiveness of flame retardants through finer process control and innovation.
(2) Compound: For PA66 with high flame retardant requirements, a single flame retardant is usually difficult to meet strict flame retardant standards. In contrast, compound flame retardants can not only significantly improve the flame retardant efficiency through the synergistic effect of a variety of flame retardant components but also reduce the total amount of addition; this approach better preserves the comprehensive performance of the material. In recent years, some emerging flame retardant strategies (such as intumescent systems and layer–by–layer assemblies) have shown outstanding application potential by promoting the formation of char layers and strengthening synergies. Therefore, the development of high–performance flame retardant PA66 based on compounding technology will become an important direction of future research.
(3) Green and environmentally friendly: Green and environmentally friendly have become an important trend in the development of flame retardant materials. With the advancement of industry technology and the increasing public awareness of health and environmental protection, halogen flame retardants are gradually facing elimination. In the future, the investigation and application of halogen–free flame retardants and environmentally friendly flame retardants will become the development focus of flame retardant PA66. To meet the growing market demand for safety, environmental protection and sustainable development, the use of these flame retardants is expected to continue increasing.
(4) Multifunctionalization: The multifunctionalization of flame retardant PA66 will become an important direction of future research. With the increasing complexity of industrial application scenarios, it is difficult for materials with single properties to meet diverse needs. For this reason, PA66 is evolving from simple flame retardant to multifunctionalization, such as combining flame retardancy with antibacterial, antistatic, UV–resistant or mechanically enhanced properties. Relevant applications include smart textiles, automotive wiring requiring durability and fire resistance, and enclosures that demand both thermal stability and EMI shielding. Therefore, the development of multifunctional flame retardant PA66 to meet the urgent needs of modern industry for high–performance, multipurpose materials will become a key research direction in the future.
(5) Additional emerging trends: In the future, the development trend of flame retardant PA66 will pay more attention to recyclability and circular economy integration, reducing the environmental burden by designing flame retardant systems that are compatible with recycling. At the same time, the widespread application of advanced characterization and simulation technologies (e.g., AI, modeling and high–throughput screening) will accelerate the prediction of flame behavior and material optimization. In addition, the rise in smart flame retardants, such as thermally triggered char formation and other stimuli–responsive systems that can dynamically enhance flame retardancy in the event of a fire, further promotes the development of efficient and adaptive flame retardant PA66. These innovative directions reflect the evolution of flame retardant technology toward sustainability, precision and functionality.

## Figures and Tables

**Table 1:** Copolymerized flame retardant PA66.

Reactive Flame Retardants	Molecular Structures	References
*Bis* (4–carboxyphenyl) phenyl phosphine oxide (BCPPO)	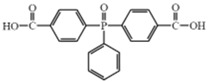	[56,57,58]
*N*–benzoic acid (ethyl–*N*–benzoic acid formamide) phosphamide (NENP)	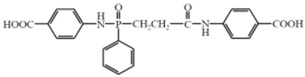	[30]
*Poly*–*N*–aniline–phenyl phosphamide (PDPPD)	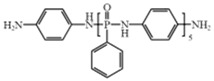	[59]
2–carboxy ethyl (phenyl) phosphinic acid (CEPPA)	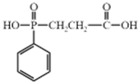	[60]
Reactive phosphorus–containing flame retardant (FR–B)	–	[61]
Reactive flame retardant (DPDA)	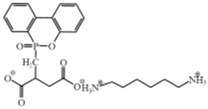	[62]
